# Mechanical Response and Low-Order Wave Propagation of Curved-Rod Compression–Torsion Coupled Metamaterials

**DOI:** 10.3390/ma19153284

**Published:** 2026-08-03

**Authors:** Wei Gao, Jun Zhu

**Affiliations:** School of Science, Lanzhou University of Technology, Lanzhou 730050, China; 18794910201@163.com

**Keywords:** compression–torsion coupling, periodic structure, spatial curved rod, equivalent stiffness, Bloch dispersion, acceleration transmissibility

## Abstract

**Highlights:**

- A spatial curved-rod architecture is proposed for compression–torsion periodic structures.
- A two-degree-of-freedom equivalent stiffness model is developed for the unit cell.
- Coupling stiffness modifies low-order dispersion and modal composition.
- Rod size and nodal mass tune the low-frequency response without local resonators.
- Numerical and experimental results support the predicted static coupling trend and the qualitative low-order dispersion characteristics.

**Abstract:**

Compression–torsion coupled metamaterials provide a geometry-driven approach for motion conversion and wave regulation. This study proposes a spatial curved-rod compression–torsion coupled metastructure and develops a reduced-order equivalent stiffness model with axial displacement and rotational angle as the generalized degrees of freedom. The equivalent stiffness matrix, derived from the curved-rod centerline geometry through a strain-energy formulation, is incorporated into a one-dimensional Bloch model to characterize low-frequency wave propagation. The results show that the off-diagonal coupling stiffness governs the compression-induced rotational response of the unit cell and significantly affects the frequencies and modal compositions of the two lowest dispersion branches. Finite element simulations and experiments validate the predicted stiffness and compression–torsion response, while acceleration transmissibility measurements of a finite periodic chain are consistent with the theoretical dispersion characteristics. The reduced-order model captures the overall behavior of the two lowest branches and clarifies the role of compression–torsion coupling stiffness in branch separation and modal composition, although the higher branch is less accurately predicted owing to the lumped inertia approximation. The proposed design provides a lightweight strategy for low-frequency wave regulation through structural coupling.

## 1. Introduction

Compression–torsion coupled metamaterials exhibit geometry-controlled axial-to-rotational motion conversion under tensile or compressive loading, with their mechanical response governed primarily by structural architecture rather than constituent material properties [1,2]. This coupling can be realized through a variety of geometrical configurations, including chiral units, inclined ligaments, helical load paths, and asymmetric connections [3], thereby enabling programmable stiffness, tunable wave propagation, and multifunctional mechanical behavior [4,5]. These capabilities have made compression–torsion coupled metamaterials attractive for applications in motion conversion, energy absorption, vibration control, and lightweight structural systems.

Various structural concepts have been proposed to achieve controllable axial-to-rotational motion conversion. Early studies mainly focused on chiral mechanical metamaterials exhibiting pronounced compression–torsion coupling [6], followed by the development of chiral and tetrachiral honeycombs [7,8], anti-chiral topologies, inclined-ligament configurations [9,10,11], and other geometry-driven architectures [12,13]. These designs have subsequently been combined with negative Poisson’s ratio effects [14,15], multistage deformation and frictional energy dissipation [16,17], as well as buckling-induced energy absorption [18]. In addition, geometric parameters have been shown to play a key role in tailoring the coupling response of three-dimensional metastructures [19]. Despite these advances, most existing architectures remain dependent on chiral lattices, inclined ligaments, or anti-chiral units. In contrast, the present study introduces a monolithic spatial curved-rod architecture that provides a simple yet highly parameterizable route for realizing lightweight compression–torsion coupled metamaterials.

Existing analytical formulations for compression–torsion coupled structures have been established using beam theory, strain-energy methods, the dummy-load method, and equivalent continuum approaches. Yang et al. [20] derived the equivalent stiffness matrix of a representative unit cell, identifying its axial, torsional, and coupling stiffness components. Subsequent studies have examined the influence of geometry, topology, and nonlinear deformation on twisting behavior, Poisson’s ratio, effective stiffness, and energy absorption [21,22,23]. However, these efforts have largely been confined to isolated unit cells or finite assemblies. In periodically assembled structures, the equivalent stiffness matrix directly enters the governing dynamic equations and therefore influences the low-order wave propagation characteristics. Nevertheless, the incorporation of the compression–torsion stiffness matrix into a periodic dynamic framework, together with the role of coupling stiffness in shaping dispersion branches and modal composition, remains insufficiently understood.

Periodic assembly enables compression–torsion coupled unit cells to support wave propagation governed by the equivalent stiffness matrix, nodal inertia, and the coupling between generalized degrees of freedom, which is commonly analyzed within the Bloch framework [24]. Existing low-frequency wave manipulation strategies are primarily based on Bragg scattering, local resonance, inertial amplification, and structural parameter tuning. Bragg scattering is inherently limited by the lattice periodicity, whereas local resonance achieves subwavelength attenuation by introducing resonant masses or compliant connections [25]. Inertial amplification has further extended low-frequency bandgap design through linkage-based mechanisms [26], inerter-inspired systems [27], seismic wave attenuation [28,29], and plate-like vibration control [30], while prestress and geometric tuning provide additional means of shifting bandgap frequencies [31]. Although highly effective, these approaches generally rely on supplementary resonant or inertial components, increasing structural complexity and reducing the advantages of lightweight structural design.

Translational–rotational coupling has also been exploited in origami-inspired and reconfigurable metamaterials. Kresling origami structures exhibit switchable torsional bandgaps and enhanced vibration attenuation through compression–torsion interactions [32,33]. Zhang et al. [34] further demonstrated that combining compression–torsion coupling with inertial amplification in a pre-twisted tubular metamaterial enables the formation of low-frequency bandgaps. Lightweight lattice structures have likewise highlighted the potential of structural coupling for vibration attenuation and impact mitigation [35,36]. Despite these advances, existing studies have primarily focused on specific structural concepts or bandgap generation mechanisms, whereas the influence of the intrinsic compression–torsion coupling stiffness on the frequencies and modal compositions of low-order dispersion branches remains insufficiently understood.

Despite these advances, existing studies have primarily focused on specific structural concepts or bandgap generation mechanisms, whereas the influence of the intrinsic compression–torsion coupling stiffness on the frequencies and modal compositions of low-order dispersion branches remains insufficiently understood [37,38,39]. Such spatial load-transfer paths have inspired a variety of bioinspired helical structures, where pitch, curvature, and winding geometry govern the overall mechanical response [40,41,42]. Inspired by these characteristics, this study proposes a spatial curved-rod compression–torsion coupled metastructure that links static axial–rotational coupling with low-order wave propagation. A reduced-order equivalent stiffness model is developed from the curved-rod centerline geometry through a strain-energy formulation and integrated into a one-dimensional Bloch framework to reveal the role of compression–torsion coupling stiffness in governing dispersion characteristics and modal composition. The proposed formulation is validated through finite element simulations, compression experiments, Bloch finite element analysis, and acceleration transmissibility measurements. Finally, the effects of rod cross-sectional size and nodal mass on the low-frequency dynamic response are investigated, demonstrating the geometric tunability of the proposed metastructure.

## 2. Theoretical Model and Equivalent Stiffness

### 2.1. Unit-Cell Configuration Design

**Figure 1:**
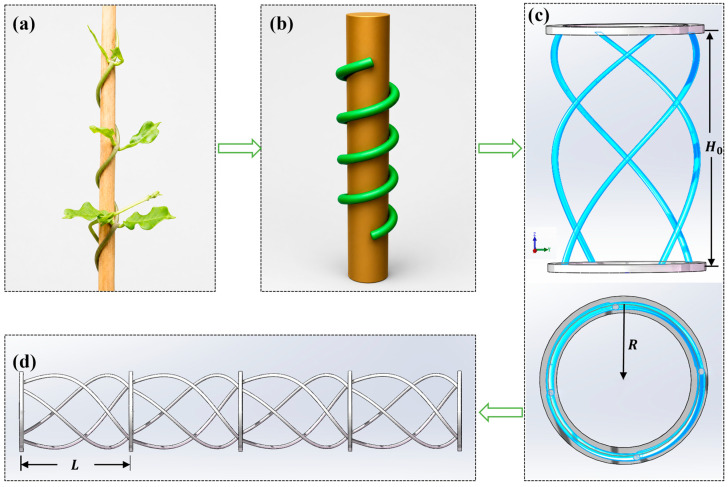
Configuration source and generation process of the compression–torsion coupled periodic structure. (**a**) Spatial winding morphology formed by climbing plants around a support; (**b**) helical curved rod abstracted from the natural winding path; (**c**) compression–torsion coupled unit cell composed of circumferentially distributed spatial curved rods and its top view; (**d**) finite chain formed by periodically assembling identical compression–torsion unit cells along the axial direction, where L denotes the axial repeat distance of one unit cell.

Figure 1 illustrates the configuration source and generation process of the proposed compression–torsion coupled structure. Inspired by the spatial winding path, the structural motif is abstracted into spatial curved rods, as shown in Figure 1c. The unit cell considered in this study consists of upper and lower end plates connected by four circumferentially distributed curved rods. These rods have identical material properties, cross-sectional dimensions, centerline geometry, and winding direction, while differing only in their initial circumferential phases. By repeatedly assembling identical unit cells along the central axis, a one-dimensional chain of compression–torsion coupled units is obtained, as shown in Figure 1d.

The spatial curved-rod unit cell consists of four smoothly wound rods distributed circumferentially, forming a continuous axial–rotational load-transfer path and ensuring structural balance. In Figure 1d, the period length L is defined as the axial repeat distance of one complete unit cell in the periodically assembled structure, namely the distance between two adjacent equivalent nodal planes. Once the unit-cell geometry is fixed, the finite periodic structure is generated by repeating the same unit cell along the axial direction with the period length L. The upper and lower end plates are assumed to be rigid, and their own deformation is neglected. Therefore, the overall deformation of the unit cell is described by two generalized degrees of freedom: the axial relative displacement u and the relative rotational angle ϕ. The generalized displacement vector d and the corresponding generalized force vector f of the unit cell are then defined as

(1) $$d=\left[\begin{array}{c} u\\ \phi \end{array}\right],\;f=\left[\begin{array}{c} F\\ M \end{array}\right]$$

where F and M denote the resultant axial force and the resultant torque, respectively. In this study, the compressive displacement is taken as positive.

### 2.2. Geometric Description of the Curved-Rod Centerline

An arbitrary curved rod in the unit cell is selected as the representative member. Its initial centerline is expressed as

(2) $$r_{0}(t)=\left[\begin{array}{c} R\cos\psi_{0}(t)\\ R\sin\psi_{0}(t)\\ H_{0}t \end{array}\right],0\leq t\leq T$$

where R is the radius of the cylindrical surface on which the rod centerline lies, $H_{0}$ is the initial axial height, t is a dimensionless centerline parameter. The axial coordinate of the initial centerline is $z=H_{0}t$, and $\psi_{0}(t)$ denotes the circumferential angular position of the rod centerline. As shown in Figure 1a, a quadratic function is adopted to describe the winding path of the curved rod along the axial direction:

(3) $$\psi_{0}(t)=at^{2}+bt+c$$

where a and b control the axial variation of the circumferential winding path, and c is the initial circumferential phase of the reference rod. The initial centerline of the j-th curved rod can then be written as

(4) $$r_{0j}\left(t\right)=\left[\begin{array}{c} \mathrm{Rcos}\left[\psi_{0}(t)+\delta_{j}\right]\\ \mathrm{Rsin}\left[\psi_{0}(t)+\delta_{j}\right]\\ H_{0}t \end{array}\right],\;\;\delta_{j}=\left(j-1\right)\frac{\pi }{2},j=1,2,3,4$$

where $\delta_{j}$ is the initial circumferential phase offset of the j-th rod. Since the four curved rods differ only in their initial phase offsets, they have the same geometric form and undergo equivalent deformation under identical end-plate motion. Therefore, the following derivation is carried out for one representative curved rod, and the contribution of all four rods is accounted for when constructing the equivalent stiffness matrix of the unit cell.

The arc-length differential of the initial centerline is given by

(5) $$ds_{0}=∥r_{0}^{\prime }(t)∥dt=v_{0}(t)dt$$

The initial curvature and torsion can be determined from the first-, second-, and third-order derivatives of the curved-rod centerline [43],

(6) $$\left\{\begin{array}{c} \kappa_{0}\left(t\right)=\frac{∥r_{0}^{\prime }(t)\times r_{0}^{\prime \prime }(t)∥}{{∥r_{0}^{\prime }(t)∥}^{3}}\\ \tau_{0}(t)=\frac{\left[r_{0}^{\prime }(t)\times r_{0}^{\prime \prime }(t)\right]\cdot r_{0}^{\prime \prime \prime }(t)}{{∥r_{0}^{\prime }(t)\times r_{0}^{\prime \prime }(t)∥}^{2}} \end{array}\right.$$

These geometric quantities are determined by R, $H_{0}$, a, and b, and are used as the initial reference state for the subsequent small-deformation strain-energy calculation.

### 2.3. Assumed-Field Kinematics and Centerline-Based Geometric Measures

Within the small-deformation range, an assumed-field kinematic approximation is adopted to relate the generalized end-plate motion to the deformation of the curved-rod centerline. This approximation can be regarded as a low-order Ritz-type displacement field rather than the exact equilibrium solution of an elastic curved rod. Since the compressive displacement is taken as positive, the current axial height of the unit cell is defined as $H(u)=H_{0}-u$. The circumferential angle of the deformed centerline is defined as $\psi (t;\phi )\;=\;\psi_{0}(t)\;+\;t\phi$, where $\psi_{0}(t)\;=\;at^{2}\;+\;bt\;+\;c$ is the initial circumferential angle defined in Equation (3) and tϕ represents the linearly interpolated end rotation from the lower plate to the upper plate. Accordingly, p(t;ϕ) is defined as the derivative of ψ(t;ϕ) with respect to t, namely $p(t;\phi )\;=\;p_{0}(t)\;+\;\phi$, where $p_{0}(t)\;=\;2at\;+\;b$. In this assumed field, the curved-rod centerline is assumed to remain on the cylindrical surface with radius R, while the axial displacement and circumferential rotation are interpolated between the lower and upper end plates. The deformed centerline of the representative curved rod is therefore assumed as

(7) $$r(t;u,\phi )=\left[\begin{array}{c} R\cos\psi (t;\phi )\\ R\sin\psi (t;\phi )\\ H(u)t \end{array}\right]$$

This assumed field satisfies the lower-end constraint and the prescribed axial–rotational motion at the upper end. It does not, however, permit independent radial motion or a fully general redistribution of bending and twisting along the rod. Given the present focus on low-order responses dominated by rigid end-plate motions, this approximation is deemed sufficient to capture the dominant axial–rotational coupling. The deformed arc-length differential, curvature, and geometric torsion are then expressed as follows

(8) $$\left\{\begin{array}{c} ds=v(t;u,\phi )dt\\ \kappa (t;u,\phi )=\frac{R\sqrt{H^{2}(u)\left[p^{4}(t;\phi )+p_{0}^{\prime 2}(t)\right]+R^{2}p^{6}(t;\phi )}}{{\left[R^{2}p^{2}(t;\phi )+H^{2}(u)\right]}^{3/2}}\\ \tau (t;u,\phi )=\frac{H(u)p(t;\phi )\left[p^{4}(t;\phi )+3p_{0}^{\prime 2}(t)\right]}{H^{2}(u)\left[p^{4}(t;\phi )+p_{0}^{\prime 2}(t)\right]+R^{2}p^{6}(t;\phi )} \end{array}\right.$$

where

(9) $$\left\{\begin{array}{c} v(t;u,\phi )=\sqrt{R^{2}p^{2}(t;\phi )+H^{2}(u)}\\ p_{0}\left(t\right)=\psi_{0}^{\prime }\left(t\right)=2at+b\\ p_{0}^{\prime }(t)=\psi_{0}^{\prime \prime }(t)=2a \end{array}\right.$$

Relative to the initial configuration, the axial centerline strain, the change in scalar curvature, and the change in geometric torsion are introduced as centerline-based geometric measures. These quantities are used to construct a reduced-order equivalent stiffness model, rather than a complete material-frame strain description of a general three-dimensional rod. These changes are defined as

(10) $$\left\{\begin{array}{c} \varepsilon (t;u,\phi )=\frac{ds-ds_{0}}{ds_{0}}=\frac{v(t;u,\phi )}{v_{0}(t)}-1\\ \Delta \kappa (t;u,\phi )=\kappa (t;u,\phi )-\kappa_{0}(t)\\ \Delta \tau (t;u,\phi )=\tau (t;u,\phi )-\tau_{0}(t) \end{array}\right.$$

By performing a first-order linearization around $\left(u,\phi )=(0,0\right)$, these centerline-based geometric measures are approximated as

(11) $$\left\{\begin{array}{c} \varepsilon (t;u,\phi )\approx \varepsilon_{u}(t)u+\varepsilon_{\phi }(t)\phi \\ \Delta \kappa (t;u,\phi )\approx \kappa_{u}(t)u+\kappa_{\phi }(t)\phi \\ \Delta \tau (t;u,\phi )\approx \tau_{u}(t)u+\tau_{\phi }(t)\phi \end{array}\right.$$

Here, $\varepsilon_{u}$, $\varepsilon_{\phi }$, $\kappa_{u}$, $\kappa_{\phi }$, $\tau_{u}$ and $\tau_{\phi }$ are sensitivity functions obtained from the linearization of the centerline geometry. This linearized kinematic relation maps the generalized end-plate displacements u and ϕ to the distributed local deformation measures along the curved rod.

### 2.4. Equivalent Stiffness Matrix

The kinematic description adopted in this work is based on the assumption that the material director frame remains approximately aligned with the Frenet–Serret frame under the considered deformation regime, thereby allowing curvature and torsion to be interpreted as effective geometric descriptors of bending and twisting behavior.

The local quantities defined above are interpreted as centerline-based geometric measures that capture the axial, curvature-related, and geometric torsion-related contributions induced by the prescribed end-plate motion. The maximum initial curvature of the rod is $0.008091\;{\mathrm{mm}}^{-1}$, corresponding to a minimum local radius of curvature of 123.60 mm. For the baseline rod diameter of 2 mm and the largest diameter of 4 mm considered, the maximum diameter-to-curvature-radius ratios are 0.0162 and 0.0324, respectively, both below 0.1 [44]. The arc length is 74.71 mm, giving length-to-diameter ratios of 37.36 and 18.68, respectively.

We estimate the ratio of the transverse shear strain energy to the bending strain energy for a representative curved rod, so that quantify the validity of neglecting transverse shear deformation. For a circular cross-section rod of diameter d and length l subjected to a transverse force P (under a three-point bending-like deformation pattern), the bending strain energy and transverse shear strain energy are approximately given by $U_{b}\approx \frac{P^{2}l^{3}}{96EI},U_{s}\approx \frac{P^{2}l}{8GA_{s}}$, where $A_{s}=A/\kappa$ is the effective shear area and κ the Timoshenko shear coefficient (for a circular cross-section, κ=10/9). The ratio of the two energy contributions thus reduces to $\frac{U_{s}}{U_{b}}=\frac{3\kappa }{2}\left(1+\nu \right){\left(\frac{d}{l}\right)}^{2}$. For the baseline rod diameter of d=2 mm, with l=74.71 mm, ν=0.38, and κ=10/9, this ratio is $U_{s}/U_{b}\approx 1.65\times 10^{-3}$. For the largest diameter of d=4 mm, the ratio is $U_{s}/U_{b}\approx 6.60\times 10^{-3}$. In both cases, the shear strain energy is more than two orders of magnitude smaller than the bending strain energy. This quantitative estimate confirms that the contribution of transverse shear deformation to the total strain energy is negligibly small, thereby supporting the use of the Euler–Bernoulli-type kinematic assumption (i.e., neglecting transverse shear deformation) in the present reduced-order model.

These results justify the straight-beam sectional stiffnesses and the neglect of transverse shear deformation as reasonable leading-order approximations. This formulation does not constitute a complete material-frame rod model; it serves to derive an approximate equivalent strain energy. Accordingly, the approximate equivalent strain energy of a single curved rod is written as

(12) $$U^{\left(1\right)}\approx \frac{1}{2}\int_{0}^{T}\left[EA(\Delta \varepsilon {)}^{2}+EI(\Delta \kappa {)}^{2}+GJ_{p}(\Delta \tau {)}^{2}\right]v_{0}(t)\;dt$$

where E and G are the effective Young’s modulus and shear modulus of the material, respectively. A, I, and $J_{p}$ denote the cross-sectional area, the second moment of area, and the polar moment of inertia of the circular rod section, respectively. Here, Δκ and Δτ are interpreted as effective curvature and torsion descriptors, respectively, rather than strict material strain measures. The formulation is interpreted as a reduced-order surrogate energy functional rather than a strict Cosserat strain-energy density. The geometric measures Δκ and Δτ are used as effective kinematic descriptors for stiffness identification under the assumed alignment of material and Frenet frames. This formulation is not intended to represent a variationally exact strain-energy density, but rather serves as a quadratic stiffness-weighted functional used for reduced-order model construction [45]. The classical sectional stiffness terms EI and GJ are used here as weighting parameters rather than strict conjugate moduli associated with material-frame bending and twisting strains. By substituting Equation (11) into Equation (12), the strain energy can be written as

(13) $$U^{\left(1\right)}\approx \frac{1}{2}K_{h}^{\left(1\right)}u^{2}+K_{c}^{\left(1\right)}u\phi +\frac{1}{2}K_{m}^{\left(1\right)}\phi^{2}$$

where the contributions of a single curved rod to the axial stiffness $K_{h}^{\left(1\right)}$, rotational stiffness $K_{m}^{\left(1\right)}$, and compression–torsion coupling stiffness $K_{c}^{\left(1\right)}$ are given by

(14) $$\left\{\begin{array}{c} K_{h}^{\left(1\right)}=\int_{0}^{T}\left[EA\varepsilon_{u}^{2}+EI\kappa_{u}^{2}+GJ_{p}\tau_{u}^{2}\right]v_{0}\left(t\right)\;dt\\ K_{m}^{\left(1\right)}=\int_{0}^{T}\left[EA\varepsilon_{\phi }^{2}+EI\kappa_{\phi }^{2}+GJ_{p}\tau_{\phi }^{2}\right]v_{0}\left(t\right)\;dt\\ K_{c}^{\left(1\right)}=\int_{0}^{T}\left[EA\varepsilon_{u}\varepsilon_{\phi }+EI\kappa_{u}\kappa_{\phi }+GJ_{p}\tau_{u}\tau_{\phi }\right]v_{0}(t)\;dt \end{array}\right.$$

The total strain energy of the unit cell is obtained by summing the energy contributions of the four rods:

(15) $$U=4U^{\left(1\right)}$$

Therefore, the equivalent stiffness matrix of the unit cell can be written as

(16) $$K_{e}=\left[\begin{array}{cc} {4K}_{h}^{\left(1\right)} & {4K}_{c}^{\left(1\right)}\\ {4K}_{c}^{\left(1\right)} & {4K}_{m}^{\left(1\right)} \end{array}\right]=\left[\begin{array}{cc} K_{h} & K_{c}\\ K_{c} & K_{m} \end{array}\right]$$

Here, $K_{h}$, $K_{m}$, and $K_{c}$ denote the equivalent axial stiffness, equivalent torsional stiffness, and compression–torsion coupling stiffness of the unit cell, respectively. The generalized force–displacement relation of the unit cell is obtained by differentiating the strain energy with respect to the generalized displacements:

(17) $$\left[\begin{array}{c} F\\ M \end{array}\right]=K_{e}\left[\begin{array}{c} u\\ \phi \end{array}\right]$$

Since the stiffness matrix is derived from the quadratic form of the elastic strain energy, its off-diagonal terms satisfy the reciprocity condition. To ensure positive elastic strain energy within the small-deformation range, the stiffness matrix should satisfy

(18) $$\left\{\begin{array}{c} K_{h}>0\\ K_{m}>0\\ K_{h}K_{m}-K_{c}^{2}>0 \end{array}\right.$$

### 2.5. Compression–Torsion Coupling Indices

To evaluate the contribution of the coupling stiffness relative to the two principal stiffness terms, a dimensionless coupling strength is defined as

(19) $$\chi =\frac{|K_{c}|}{\sqrt{K_{h}K_{m}}}$$

when the stiffness matrix satisfies the positive-definiteness condition, χ<1. A larger value of χ indicates a more significant contribution of the compression–torsion coupling stiffness relative to the axial and torsional stiffnesses [46].

For the free-torsion boundary condition, no external torque is applied at the end plate, i.e., M=0. From the generalized force–displacement relation, one obtains

(20) $$K_{c}u+K_{m}\phi =0$$

Therefore, the free-torsion coupling coefficient is then defined as

(21) $$\eta =\frac{\phi }{u}=-\frac{K_{c}}{K_{m}}$$

Here, η represents the end rotation induced by a unit axial displacement under the free-torsion condition, and its absolute value characterizes the magnitude of the compression-induced rotation per unit axial displacement.

## 3. Dynamic Model and Bloch Dispersion Relation of the One-Dimensional Periodic Chain

### 3.1. Equivalent Model of the One-Dimensional Periodic Chain

Based on the periodically assembled unit-cell configuration shown in Figure 1d, the structure is idealized as a one-dimensional discrete chain along the z-axis. Each compression–torsion coupled unit cell connects two adjacent rigid end-plate planes, and these planes are treated as equivalent discrete nodes in the reduced-order dynamic model. The nodal spacing is equal to the axial repeat distance L of one unit cell, as defined in Section 2.1. The generalized displacement vector $q_{n}$ of the n-th node is defined as

(22) $$q_{n}=\left[\begin{array}{c} u_{n}\\ \phi_{n} \end{array}\right]$$

where $u_{n}$ is the axial displacement of the n-th node and $\phi_{n}$ is its rotation about the central axis. For the unit cell connecting the n-th and (n+1)-th nodes, the generalized deformation vector $\Delta q_{n}$ is given by

(23) $$\Delta q_{n}=q_{n+1}-q_{n}$$

Based on the generalized deformation between adjacent nodes and the equivalent stiffness matrix $K_{e}$ of the unit cell, the elastic potential energy stored in the unit cell connecting nodes n and n+1 can be expressed as

(24) $$\Pi_{n}=\frac{1}{2}{\left(q_{n+1}-q_{n}\right)}^{T}K_{e}\left(q_{n+1}-q_{n}\right)$$

The off-diagonal stiffness terms introduce a cross-coupling between the relative axial displacement and the relative rotation in the unit-cell energy, and therefore enter the discrete dynamic equations of the periodic structure as coupling stiffness terms.

### 3.2. Bloch Eigenvalue Problem and Dispersion Relation

A low-order lumped inertia approximation is introduced for each equivalent node, with the generalized nodal coordinates being the axial displacement u and the rotation ϕ about the central axis. The kinetic energy of the n-th equivalent node is approximated as $T_{n}=\frac{1}{2}m{\dot{u}}_{n}^{2}+\frac{1}{2}J_{m}{\dot{\phi }}_{n}^{2}$, where m is the equivalent nodal mass and $J_{m}$ is the equivalent polar rotational inertia about the central axis. The equivalent nodal inertia matrix is then given by

(25) $$M_{0}=\left[\begin{array}{cc} m & 0\\ 0 & J_{m} \end{array}\right]$$

This diagonal inertia matrix should be interpreted as a low-order lumped approximation rather than a complete consistent mass matrix of the three-dimensional curved-rod structure. A fully consistent mass matrix would require integrating the distributed kinetic energy of the curved rods under the same assumed-field kinematics, which is beyond the scope of the present two-degree-of-freedom Bloch model.

(26) $$M_{0}{\ddot{q}}_{n}+K_{e}\left(2q_{n}-q_{n+1}-q_{n-1}\right)=0$$

For an infinite periodic structure, the Bloch wave solution is assumed in the form

(27) $$q_{n}\left(t\right)=\hat{q}e^{i\left(knL-\omega t\right)},\;\;\hat{q}=\left[\begin{array}{c} \hat{u}\\ \hat{\phi } \end{array}\right]$$

where k is the Bloch wavenumber, ω is the angular frequency, respectively. Substituting the Bloch wave solution into the discrete equations of motion gives the generalized eigenvalue problem:

(28) $$\left[\lambda (k)K_{e}-\omega^{2}M_{0}\right]\hat{q}=0$$

where

(29) $$\lambda (k)=2(1-coskL)=4{sin}^{2}\frac{kL}{2}$$

The condition for the existence of nonzero eigen-amplitudes is

(30) $$det\left[\lambda (k)K_{e}-\omega^{2}M_{0}\right]=0$$

After expansion, the following quadratic equation with respect to $\omega^{2}$ is obtained:

(31) $$mJ\omega^{4}-\lambda (JK_{h}+mK_{m})\omega^{2}+\lambda^{2}(K_{h}K_{m}-K_{c}^{2})=0$$

Therefore, the two low-order dispersion branches can be expressed as

(32) $$\left\{\begin{array}{c} \omega_{1}^{2}(k)=\frac{\lambda (k)}{2mJ}\left[JK_{h}+mK_{m}-\sqrt{\left(JK_{h}-mK_{m}{)}^{2}+4mJK_{c}^{2}\right.}\right]\\ \omega_{2}^{2}(k)=\frac{\lambda (k)}{2mJ}\left[JK_{h}+mK_{m}+\sqrt{\left(JK_{h}-mK_{m}{)}^{2}+4mJK_{c}^{2}\right.}\right] \end{array}\right.$$

where $\omega_{1}^{2}(k)$ and $\omega_{2}^{2}(k)$ denote the lower and upper branches, respectively. The corresponding frequencies are given by

(33) $$\left\{\begin{array}{c} f_{1}(k)=\frac{\omega_{1}(k)}{2\pi }\\ f_{2}(k)=\frac{\omega_{2}(k)}{2\pi } \end{array}\right.$$

For the one-dimensional periodic structure, only the first irreducible Brillouin zone, ($0\leq k\leq \frac{\pi }{L}$), is considered. The above dispersion relation shows that the compression–torsion coupling stiffness $K_{c}$ enters the two low-order branches through the eigenvalue problem. Therefore, the static stiffness matrix of the unit cell not only determines the compression–torsion response of an individual unit cell, but also affects the low-order wave propagation characteristics of the periodic structure.

### 3.3. Validation of the Lumped Inertia Approximation

In the reduced-order Bloch model presented in Section 3.2, the inertia of each equivalent node is approximated by a diagonal lumped mass matrix $M_{0}=\mathrm{diag}\left(m,J_{m}\right)$, where m and $J_{m}$ are obtained from the finite element mass properties (Table 1). To assess the validity of this simplification, a Guyan condensation [47] is performed on the full three-dimensional finite element model of the unit cell.

The master degrees of freedom are chosen as the axial displacement u and the rotational angle ϕ about the central axis of the upper end plate, consistent with the generalized coordinates of the reduced-order model. The slave degrees of freedom comprise all remaining degrees of freedom of the finite element model. Following the standard Guyan reduction procedure, the full stiffness and mass matrices are partitioned as

$$\left[\begin{array}{cc} K_{mm} & K_{ms}\\ K_{sm} & K_{ss} \end{array}\right]\left[\begin{array}{c} u_{m}\\ u_{s} \end{array}\right]=\left[\begin{array}{c} F_{m}\\ 0 \end{array}\right],\left[\begin{array}{cc} M_{mm} & M_{ms}\\ M_{sm} & M_{ss} \end{array}\right]\left[\begin{array}{c} u_{m}\\ u_{s} \end{array}\right]=\left[\begin{array}{c} 0\\ 0 \end{array}\right]$$

where the subscripts m and s denote master and slave degrees of freedom, respectively. The slave displacements are expressed in terms of the master displacements through the static condensation relation $u_{s}=-K_{ss}^{-1}K_{sm}u_{m}$, yielding the transformation matrix $T=\left[\begin{array}{c} -K_{ss}^{-1}K_{sm}\\ I \end{array}\right]$. The reduced stiffness and mass matrices are then obtained as $K_{\mathrm{cond}}=T^{T}K_{\mathrm{FE}}T,M_{\mathrm{cond}}=T^{T}M_{\mathrm{FE}}T$. For the baseline configuration (d=2 mm), the condensed mass matrix is obtained as $M_{\mathrm{cond}}=\left[\begin{array}{cc} m_{11} & m_{12}\\ m_{12} & m_{22} \end{array}\right]=\left[\begin{array}{cc} 4.3\times 10^{-3} & 5\times 10^{-7}\\ 5\times 10^{-7} & 2.5\times 10^{-6} \end{array}\right]$, where the units of $m_{11}$, $m_{12}$, and $m_{22}$ are kg, kg·m, and kg·m^2^, respectively. The off-diagonal term $m_{12}$ represents the translational–rotational inertial coupling. Its magnitude relative to the diagonal terms is $\frac{|m_{12}|}{\sqrt{m_{11}m_{22}}}=\frac{5\times 10^{-7}}{\sqrt{\left(4.3\times 10^{-3}\right)\left(2.5\times 10^{-6}\right)}}\approx 0.5\%$.

This small value confirms that the inertial coupling between axial translation and rotation about the central axis is secondary and can be neglected without introducing significant error. The diagonal lumped approximation is therefore justified for the inertial coupling aspect.

However, comparison of the diagonal entries reveals quantitative differences. The condensed axial inertia $m_{11}=4.3\times 10^{-3}\;\mathrm{kg}$ is approximately 11.6% smaller than the lumped value $m=4.866\times 10^{-3}\;\mathrm{kg}$ used in the original model, while the rotational inertia $m_{22}=2.5\times 10^{-6}\;\mathrm{kg}\cdot m^{2}$ differs by only about 0.8% from the lumped value $J_{m}=2.48\times 10^{-6}\;\mathrm{kg}\cdot m^{2}$. This discrepancy in the axial inertia term arises because the lumped model assigns all mass of the curved rods to the nodal planes, whereas in the actual dynamic response, only a fraction of the rod mass participates effectively in the axial inertial motion due to the simultaneous bending deformation of the curved rods.

### 3.4. Modal Composition Index

A dimensionless modal amplitude ratio is defined from the Bloch eigenvector as $|L\hat{\phi }/\hat{u}|$, where $L\hat{\phi }$ converts the rotational amplitude into an equivalent length scale for direct comparison with the axial displacement amplitude $\hat{u}$. Larger values of this ratio indicate a more pronounced rotational component in the corresponding branch, while smaller values indicate a more dominant axial component. This index is used to assist in identifying the modal composition of the low-order dispersion branches and will be examined alongside the theoretical dispersion curves and Bloch finite-element mode shapes.

## 4. Finite Element and Experimental Validation

### 4.1. Finite Element Model and Stiffness Extraction

**Figure 2:**
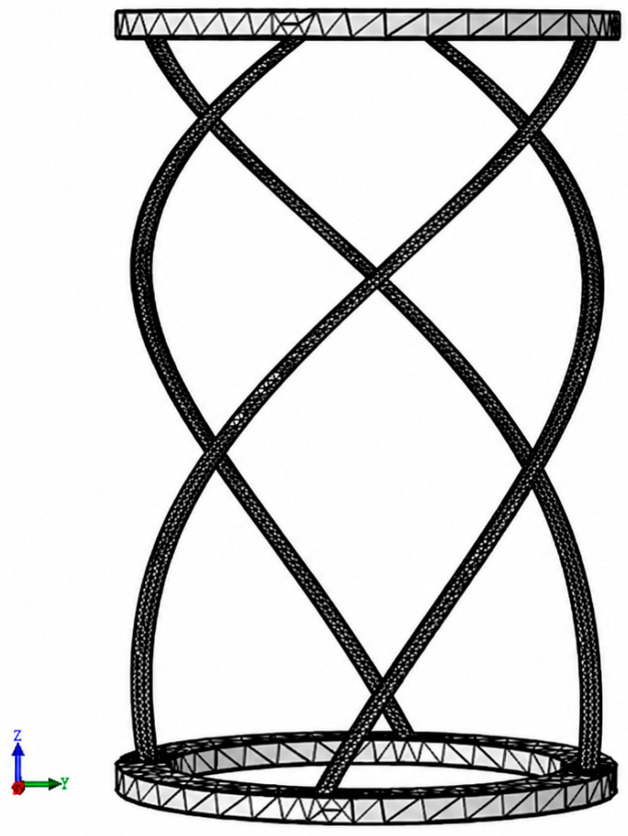
Finite element model and mesh discretization of the unit cell.

The equivalent stiffness matrix of the unit cell is validated by a three-dimensional finite element model established in COMSOL Multiphysics 6.3, as shown in Figure 2. The lower end plate is fixed, and the upper end plate is loaded with either an axial displacement or a rotational boundary condition about the central axis. The total axial reaction force $F^{\mathrm{FE}}$ and reaction torque $M^{\mathrm{FE}}$ about the central axis are extracted from the upper end plate during the calculation.

The main parameters used in this study are summarized in Table 1.

According to the generalized force–displacement relationship of the unit cell given in Equation (18), the three stiffness parameters in the equivalent stiffness matrix can be identified through two independent loading cases.

The first loading case corresponds to axial displacement loading. An axial displacement u=Δu is applied to the upper end plate, while its rotation about the z-axis is constrained, i.e., ϕ=0. In this case,

(34) $$F=K_{h}\Delta u,\;\;M=K_{c}\Delta u$$

Therefore, $K_{h}$ and $K_{c}$ can be extracted from the slopes of the (F−u) and (M−u) curves, respectively.

The second loading case corresponds to end-rotation loading. A relative rotation ϕ=Δϕ about the z-axis is applied to the upper end plate, while its axial translation is constrained, i.e., u=0. In this case,

(35) $$F=K_{c}\Delta \phi ,\;\;M=K_{m}\Delta \phi$$

Accordingly, $K_{c}$ and $K_{m}$ can be extracted from the slopes of the (F−ϕ) and (M−ϕ) curves, respectively. Since the stiffness matrix is derived from the quadratic form of the elastic strain energy, the coupling stiffness $K_{c}$ obtained from the two loading paths should be at the same numerical level. This reciprocity can also be used as a criterion for assessing the reliability of the finite element stiffness extraction.

To ensure the reliability of the finite element results, a mesh convergence study was performed using three progressively refined meshes for the baseline configuration (d=2 mm). For each mesh, the equivalent stiffness parameters ($K_{h}$, $K_{c}$, $K_{m}$) and the Bloch eigenfrequencies at the Brillouin zone boundary (kL/π=1) were extracted. Table 2 summarizes the convergence results.

The changes in $K_{h}$, $K_{c}$, $K_{m}$, and $f_{2}$ between the baseline and finest meshes are all within 3.7%, confirming good convergence. The low-frequency branch $f_{1}$ shows a larger change of 12.5%, which is attributed to its mode shape involving substantial bending deformation of the curved rods, making it more sensitive to mesh resolution. Considering the computational cost of the finest mesh (590, 246 DOFs, 846 s), the baseline mesh offers a balanced compromise between accuracy and efficiency and was therefore used for all subsequent finite element analyses.

The shear modulus was calculated from the measured Young’s modulus E and Poisson’s ratio ν as G=E/[2(1+ν)]. The equivalent nodal mass and polar rotational inertia were obtained from the finite element mass properties in COMSOL and assigned to the axial translational and rotational degrees of freedom in the discrete Bloch model, respectively.

The finite element model was established in COMSOL Multiphysics using the Solid Mechanics module. The structure was discretized using three-dimensional tetrahedral solid elements, and the displacement field was approximated using quadratic Lagrange interpolation. Separate mesh controls were applied to the lower end plate, curved rods, and upper end plate. A finer mesh was used for the curved rods, whereas relatively coarser meshes were used for the end plates. The detailed mesh parameters, including the maximum element sizes in the curved rods and end plates, are summarized in Table 1. For the baseline rod diameter of 2 mm, the maximum element size adopted for the curved rods corresponds to approximately four elements across the rod diameter. The resulting unit-cell finite element model contained 142,998 degrees of freedom.

### 4.2. Theoretical–Finite Element Comparison of the Equivalent Stiffness Matrix

**Figure 3:**
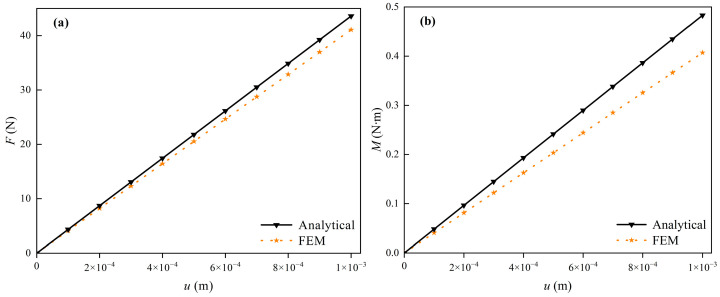
Analytical and finite element predictions under prescribed axial displacement loading: (**a**) axial reaction force–displacement curve; (**b**) reaction torque–displacement curve.

Figure 3 compares the theoretical predictions with the finite element results under axial displacement loading. Figure 3a and Figure 3b show the axial reaction force–displacement and reaction torque–displacement curves, respectively. Within the small-deformation range, both quantities vary approximately linearly with the prescribed axial displacement. The slopes of these curves yield $K_{h}$ and $K_{c}$ values.

**Figure 4:**
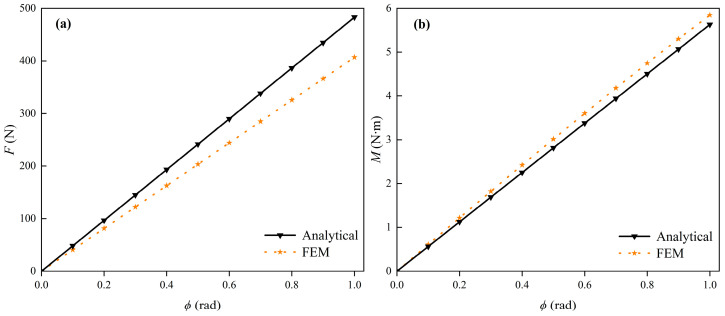
Analytical and finite element predictions under prescribed end rotation loading: (**a**) axial reaction force–rotation curve; (**b**) reaction torque–rotation curve.

Figure 4 compares the theoretical predictions with the finite element results under end-rotation loading. Figure 4a and Figure 4b show the axial reaction force–rotation and reaction torque–rotation curves, respectively. Under this loading condition, both quantities also vary approximately linearly with the prescribed rotation.

Table 3 summarizes the theoretical predictions and the stiffness values extracted from the finite element simulations. The theoretical model reasonably predicts the axial stiffness $K_{h}$ and torsional stiffness $K_{m}$. For the coupling stiffness $K_{c}$, the predicted value shows some deviation from the FE result but remains consistent in magnitude and trend. This discrepancy is mainly attributed to the assumed-field kinematic approximation, the use of centerline-based geometric measures in the energy model, and local deformation in the connection regions of the three-dimensional solid model.

### 4.3. Validation of the Assumed-Field Centerline Approximation

The accuracy of the assumed-field kinematic approximation used in Equation (7) is further evaluated by comparing the deformed centerline of a representative curved rod predicted by Equation (7) with that extracted from the three-dimensional finite element model. Two loading cases are considered for two rod diameters: an axial displacement of u=2 mm with ϕ=0, and an end rotation of ϕ=0.1 rad with u=0. For each case, the deformed centerline from the finite element model is obtained by superimposing the computed displacement components onto the initial coordinates at 20 equally spaced sampling points along the rod. The corresponding assumed-field centerline is calculated from Equation (7) under the same prescribed end motion.

The deviation between the FE-extracted and assumed-field centerlines is evaluated using three normalized indices: the root-mean-square deviation $E_{\mathrm{RMS}}/L$, the maximum spatial deviation $E_{\max}/L$, and the maximum radial deviation $E_{r,\max}/R$. The first two quantify the average and maximum centerline deviations, respectively, while the third assesses the validity of the constant-radius assumption in Equation (7), where L is the period length and R is the initial cylindrical radius of the curved-rod centerline.

As shown in Table 4, the assumed-field centerline captures the overall trend of the FE-extracted centerline under both loading cases. The normalized RMS deviations are 4.6% under axial displacement and 3.8% under end rotation, with corresponding maximum deviations of 6.3% and 4.2%. The maximum radial deviation remains below 8.1%, confirming that the constant-radius assumption introduces a measurable but limited error. Larger local deviations occur near the rod–plate connections, where local bending and radial motion are more pronounced. These local effects, together with the centerline-based curvature and torsion measures, partly explain the discrepancy between the analytical and FE stiffness results discussed in Section 4.2.

**Figure 5:**
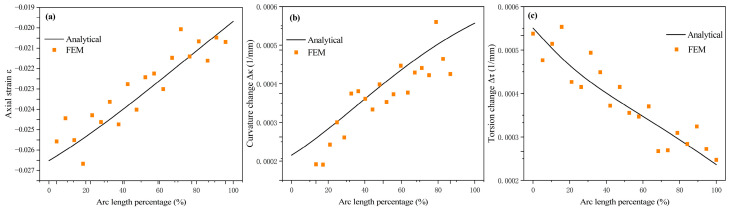
Comparison of the analytical predictions and finite element results for the distributions of (**a**) axial strain ε; (**b**) curvature change Δκ; and (**c**) torsion change Δτ, along the normalized arc length under the representative loading condition u=2 mm and ϕ=0. The finite element data are obtained by projecting the displacement field onto the deformed centerline sampled at 20 equally spaced points, following the same procedure as that used in Table 4.

The geometric deviations reported in Table 4 reflect the accuracy of the assumed centerline representation, which serves as the geometric basis for the kinematic mapping defined in Equation (7). Since the strain measures (ε, κ, and τ) are computed directly from this mapping, any deviation in the assumed centerline representation will propagate into the evaluation of the strain field. To assess the impact of these geometric deviations on the strain-level response, the axial strain, curvature variation, and torsion distributions are further examined under a representative loading condition (u = 2 mm, ϕ = 0). Under this loading case, the field responses are evaluated using both the finite element model and the analytical formulation (Figure 5).

Good agreement is observed between the analytical predictions and finite element results for axial strain, curvature variation, and torsion distributions under the representative loading case. The consistency in ε, κ, and τ distributions indicates that the proposed kinematic approximation is capable of accurately reproducing the strain-level response underlying the strain-energy formulation. These results indicate that the proposed kinematic approximation is capable of capturing the main features of the strain-level response under the considered deformation regime. The observed geometric deviations introduce only limited influence on the strain measures governing the reduced-order formulation.

### 4.4. Finite Element Validation of the Free-Torsion Coupling Coefficient

**Figure 6:**
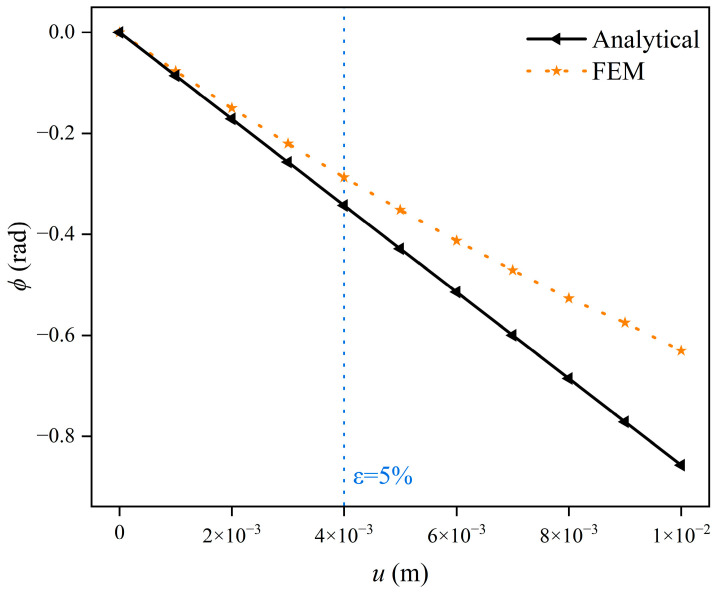
Compression displacement–rotation response under free-torsion boundary conditions: comparison between analytical and finite element results.

The free-torsion response of the unit cell under axial compression is further validated using the parameters extracted from finite element simulations. As shown in Figure 6, the upper end-plate rotation varies approximately linearly with the compressive displacement within the small-deformation range, and the theoretical model agrees well with the finite element result, confirming that the free-torsion coupling coefficient η reasonably characterizes the compression-induced rotational capability.

### 4.5. Specimen Fabrication and Experimental Validation

#### 4.5.1. Specimen Fabrication and Material Characterization

**Figure 7:**
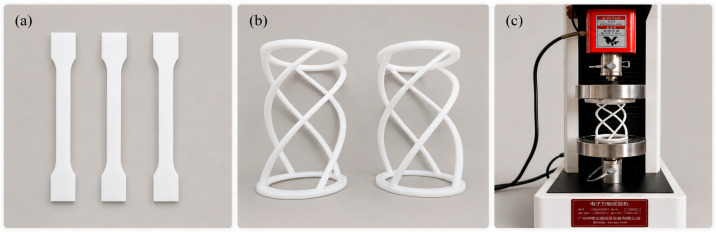
Printed specimens and experimental setup: (**a**) standard tensile specimens; (**b**) compression–torsion coupled structural specimens; (**c**) axial compression test setup.

Two types of specimens were fabricated: standard tensile specimens for identifying the elastic modulus of the printed ABS material, and compression–torsion coupled unit-cell specimens for validating the axial and rotational responses of the proposed structure. This separation allows material-parameter identification and structural-response validation to be performed independently while maintaining consistent material and printing parameters across theoretical, finite element, and experimental comparisons. The specimens were manufactured using a P1S printer with fused deposition modeling, using ABS filament, a 0.4 mm nozzle, 0.2 mm layer thickness, 45° printing orientation, and 100% infill density. Three tensile specimens and two structural specimens were fabricated. Figure 7 shows the printed specimens and the experimental setup.

**Figure 8:**
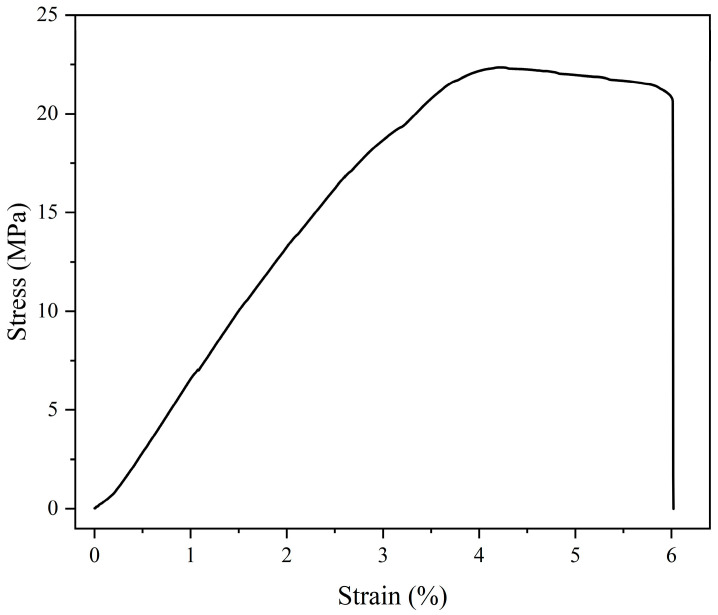
Engineering stress–strain responses of the 3D-printed standard tensile specimens.

The standard tensile specimens were tested under displacement-controlled loading at a loading rate of 5 mm/min. The load and displacement were recorded, and the engineering stress–strain curves were calculated. Figure 8 shows the engineering stress–strain curves of the printed ABS specimens. Based on linear fitting of the initial elastic region, the effective Young’s modulus of the printed material was determined to be E=0.718 GPa. This effective modulus was used in the subsequent theoretical calculations and finite element models.

#### 4.5.2. Experimental Validation of Compression–Torsion Coupling

After obtaining the effective elastic modulus of the printed material, axial compression tests were conducted on the compression–torsion coupled structural specimens. During the tests, the axial load and compressive displacement were recorded, and the rotation of the upper end plate was extracted using an image-based measurement method. For the image-based rotation measurement, two markers were attached to the upper end plate before testing. The deformation process was recorded by a camera placed perpendicular to the observation plane. The pixel-to-length scale was calibrated using the known specimen dimension. The coordinates of the two markers were extracted from the recorded images, and the rotation of the upper end plate was calculated from the change in the inclination angle of the line connecting the two markers. The measured rotation angles were converted into radians for comparison with the theoretical and finite element results. In this way, the load–displacement relationship and the rotation–displacement relationship of the structure were obtained.

**Figure 9:**
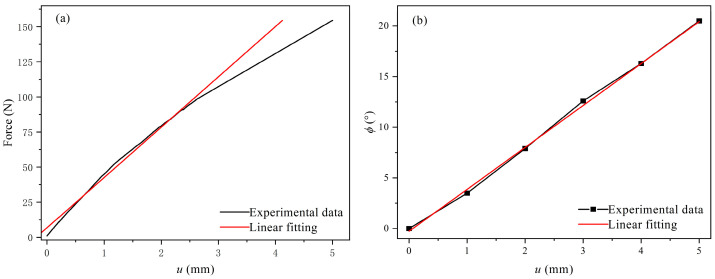
Experimental axial compression responses of the compression–torsion coupled specimens: (**a**) axial load versus compression displacement; (**b**) end rotation versus compression displacement. The experimental data represent the mean values of three repeated measurements.

Figure 9a shows the axial load–compressive displacement curves of the structural specimens. Within the initial small-deformation range, the axial load increases approximately linearly with the compressive displacement. By fitting this linear region, the experimentally identified equivalent axial stiffness is obtained as $K_{h}^{exp}=3.93\times 10^{4}\;N/m$. Figure 9b shows the variation in the end rotation with the compressive displacement. The experimental results indicate that the rotation of the upper end plate increases approximately linearly with the compressive displacement, confirming that the structure can generate a stable rotational response about the central axis under axial compression. Based on the initial linear region of the rotation–displacement curve, the experimental free-torsion coupling coefficient was obtained from the fitted slope as

(36) $$\eta^{exp}=\frac{∆\phi^{exp}}{∆u^{exp}}$$

where $∆\phi^{exp}$ and $∆u^{exp}$ denote the experimentally measured compression displacement and end rotation, respectively. For comparison with the theoretical and finite element results, the experimentally measured rotation angles were uniformly converted into radians.

Table 5 summarizes the theoretical, finite element, and experimental results for the equivalent axial stiffness $K_{h}$ and the free-torsion coupling coefficient η. The three sets of results are generally within a comparable numerical range. For $K_{h}$, the differences among the three results are relatively small, confirming that the theoretical model captures the axial load-bearing behavior in the small-deformation regime. For η, the finite element and experimental results agree well, while the theoretical model captures the trend and order of magnitude. The discrepancy between the theoretical and experimental results is mainly attributed to the low-order kinematic assumption, local deformation in the rod–plate connection regions, manufacturing deviations, and measurement errors.

Based on the finite element and experimental validation, the equivalent stiffness matrix of the unit cell is further employed in the low-order wave propagation analysis of the periodic structure.

## 5. Wave Propagation Characteristics and Parametric Regulation

### 5.1. Bloch Dispersion Relations

The derived equivalent stiffness and nodal inertia parameters of the baseline configuration are substituted into the one-dimensional Bloch dispersion model. The baseline curved-rod centerline has geometric parameters a=0.29, b=0.86, and c=0.5.

**Figure 10:**
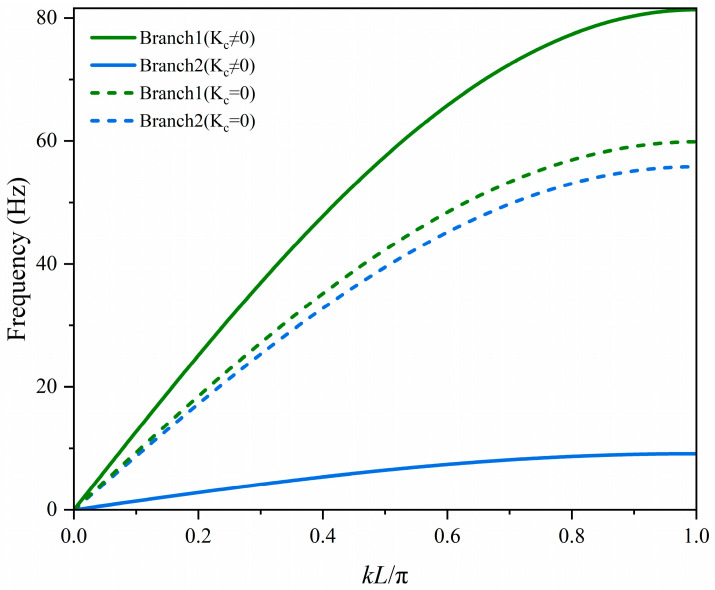
Effect of compression–torsion coupling stiffness on the analytical dispersion branches of the baseline configuration.

To isolate the effect of the compression–torsion coupling stiffness on the dispersion branches, a theoretical reference case is introduced in which $K_{h}$, $K_{m}$, the equivalent nodal mass, and the equivalent rotational inertia are kept unchanged while only the coupling stiffness is set to $K_{c}=0$. This case does not correspond to an actual structural configuration and is used solely to identify the contribution of the coupling stiffness term. Figure 10 compares the dispersion curves for the two cases.

Figure 10 shows that the compression–torsion coupling stiffness alters the frequency positions of the two low-order dispersion branches. When $K_{c}=0$, the axial displacement and rotation about the central axis are decoupled, and the two branches correspond primarily to axial-dominated and torsion-dominated propagation, respectively. When the actual coupling stiffness $K_{c}$ of the baseline configuration is introduced, the two branches become separated, indicating that axial translation and rotation no longer participate independently but instead form coupled compression–torsion propagation branches.

### 5.2. Validation by Bloch Finite Element Analysis

**Figure 11:**
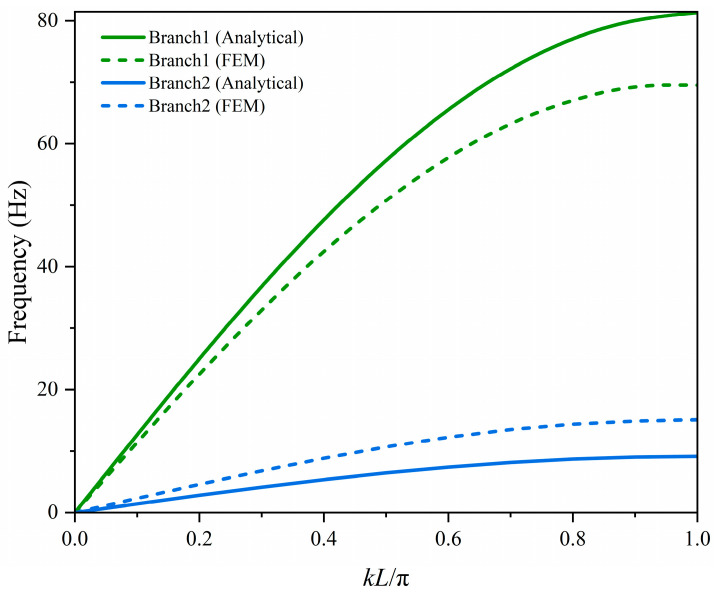
Analytical dispersion curves and Bloch finite element results for the baseline configuration.

Figure 11 compares the dispersion curves of the baseline configuration obtained from the one-dimensional equivalent model and the three-dimensional Bloch finite element model. The one-dimensional model captures the overall trends of the two low-order branches and the branch separation characteristics. However, because the theoretical model retains only two degrees of freedom per node (axial displacement and rotation about the central axis), whereas the three-dimensional finite element model includes local bending of the curved rods, deformation of the connection regions, and higher-order local modes, certain discrepancies in the predicted frequencies are observed.

**Figure 12:**
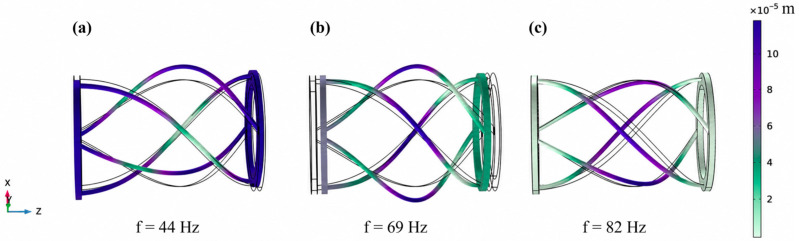
Representative displacement amplitude fields from Bloch finite element analysis: (**a**) 44 Hz; (**b**) 69 Hz; (**c**) 82 Hz. The color bar indicates displacement amplitude.

Figure 12 shows the displacement-amplitude distributions at several representative frequency points obtained from the Bloch finite element analysis. The response patterns of the spatial curved rods and end plates differ at different frequencies. The structural motion involves the combined participation of axial displacement, rotation about the central axis, and local bending of the curved rods. This indicates that the low-order propagation branches are not associated with purely axial or purely torsional responses, but instead exhibit evident compression–torsion coupling characteristics.

Table 6 lists the average errors and average relative errors of the two low-order branches. The theoretical model captures the frequency trend of Branch 1 reasonably well, with an average relative error of 14.7%, while Branch 2 shows a larger deviation of 39.9%. This indicates that the present two-degree-of-freedom model is more suitable for capturing the low-order coupling trend than for providing high-accuracy predictions across all branches.

The relatively large error in Branch 2 is mainly attributed to the reduced-order inertial approximation. In the present Bloch model, the distributed inertia of the curved rods and possible local deformation modes of the three-dimensional structure are not explicitly retained. Therefore, the diagonal nodal inertia matrix should be regarded as a low-order lumped approximation rather than a complete consistent mass matrix. Nevertheless, the model still provides a useful interpretation of the influence of compression–torsion coupling on the low-order dispersion characteristics.

### 5.3. Modal Composition of the Dispersion Branches

**Figure 13:**
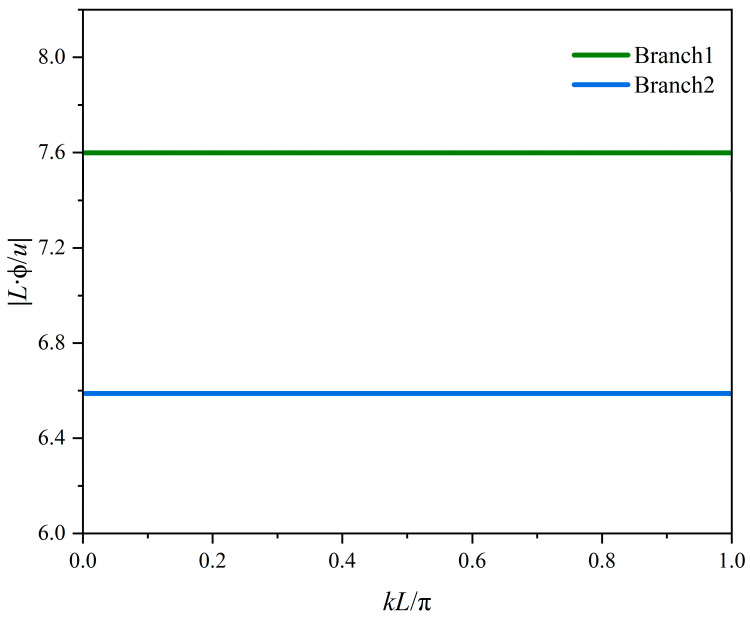
Dimensionless modal amplitude ratios for the two analytical dispersion branches of the baseline configuration.

To further identify the relative participation of axial motion and rotation about the central axis in the two theoretical dispersion branches, the dimensionless modal amplitude ratio $\left|\frac{L\hat{\phi }}{\hat{u}}\right|$, defined in Section 3.3, is used to analyze the Bloch eigenvectors of the baseline configuration. Figure 13 presents the modal amplitude ratios for Branch 1 and Branch 2. Branch 1 is dominated by rotation, while Branch 2 is dominated by axial motion.

As shown in Figure 13, the modal amplitude ratios of the two branches remain relatively stable within the first irreducible Brillouin zone. The ratio of Branch 1 is consistently higher than that of Branch 2, indicating a more pronounced rotational contribution in Branch 1 and a more dominant axial contribution in Branch 2. Since both ratios are nonzero, neither branch is purely axial or purely torsional; instead, both are coupled compression–torsion propagation branches.

Combining the observations from Figure 10 and Figure 13, it can be seen that the compression–torsion coupling stiffness $K_{c}$ not only changes the frequency positions of the two low-order dispersion branches, but also promotes the coupled participation of axial motion and rotation about the central axis in the propagation modes. Therefore, a correspondence is established between the static compression–torsion coupling characteristics of the unit cell and the modal composition of the low-order wave propagation in the periodic structure.

### 5.4. Experimental Validation of Frequency Response

**Figure 14:**
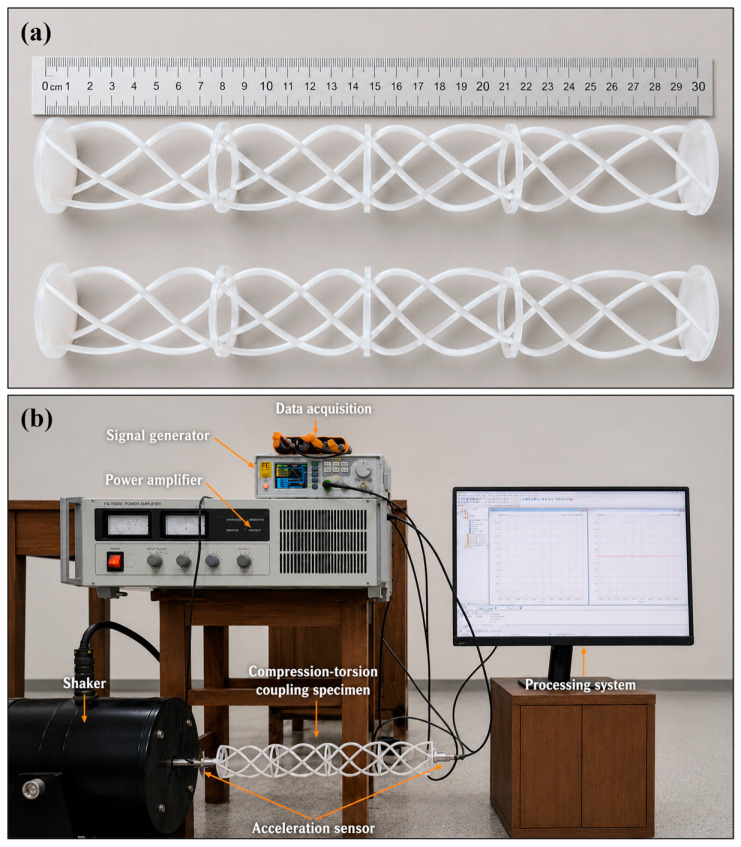
Finite periodic specimen and vibration testing system: (**a**) finite periodic compression–torsion coupling specimens; (**b**) experimental setup for acceleration transmissibility measurement.

The finite periodic specimen and experimental setup are shown in Figure 14. In the frequency-domain finite element analysis, an axial displacement excitation was applied at the left end of the structure, while the right end was kept as a free boundary. The mass-loading effect of the accelerometers was accounted for by adding two 3.2 g point masses at the locations corresponding to the sensor attachment positions (i.e., at the input and output ends of the specimen). The analyses were performed with a constant loss factor damping model, where the loss factor was set to η=0.03, which is a representative value for 3D-printed ABS material. The frequency step was refined to 2 Hz to resolve the attenuation feature accurately. The axial acceleration responses at the input and output ends were then extracted. The acceleration transmissibility is defined as

(37) $$T_{a}\left(f\right)=20{log}_{10}\left|\frac{a_{out}(f)}{a_{in}(f)}\right|$$

where $a_{in}(f)$ and $a_{out}(f)$ are the acceleration amplitudes at the input and output ends at frequency f, respectively.

In the experiment, the specimen was excited at the left end using a SA-JZ series modal shaker driven by a SA-PA series power amplifier (Wuxi Shiao Technology Co., Ltd., Wuxi, China), with a sinusoidal displacement excitation of 1 mm amplitude from 10 Hz to 200 Hz in 10 Hz steps. At each frequency step, the excitation was maintained for 3 s to ensure steady-state response before recording. Within the 60–80 Hz range where the transmission rate attenuation is more significant, the step size is adjusted to 2 Hz in order to better observe the attenuation characteristics. The input and output accelerations were acquired by SAED0005B IEPE-type accelerometers (Wuxi Shiao Technology Co., Ltd., Wuxi, China; sensitivity: 100 mV/g at 20 ± 5 °C) via a SA1804A four-channel dynamic signal analyzer (Wuxi Shiao Technology Co., Ltd., Wuxi, China; 24-bit A/D, 128 kHz/channel maximum sampling rate). Data acquisition and signal processing were performed using the DASP (Data Acquisition & Signal Processing) software (Version V2026; China Orient Institute of Noise & Vibration, Beijing, China). The sampling frequency was set to 2000 Hz. A Hanning window with 75% overlap was applied, and each measurement was repeated three times to assess repeatability.

**Figure 15:**
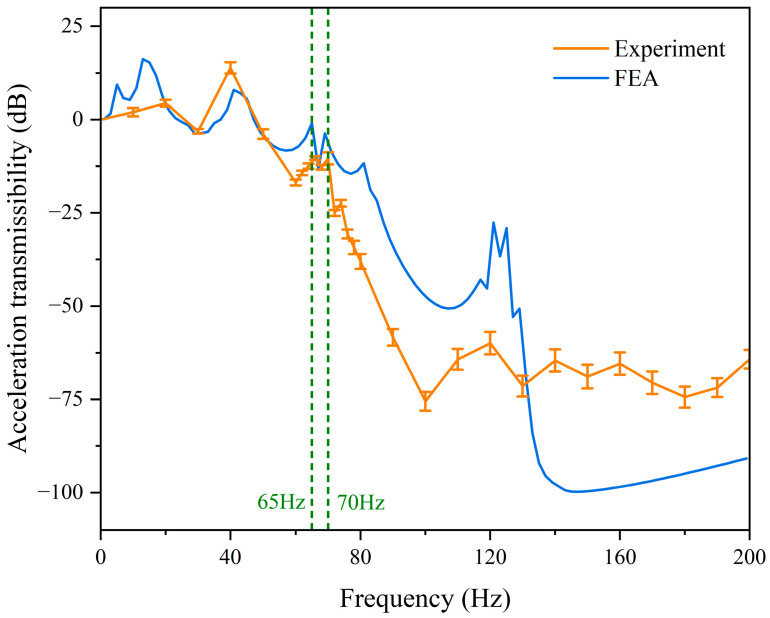
Comparison of acceleration transmissibility obtained from finite element prediction and experimental measurement. The experimental data represent the mean values of three repeated measurements, error bars indicate ±3.2 standard deviation (SD).

Figure 15 compares the finite element transmissibility the experimental data. Both curves exhibit a pronounced attenuation in the frequency range of approximately 65–70 Hz, with the FEA prediction showing the onset near 65 Hz and the experimental measurement near 70 Hz. The error bars in Figure 15 show that the standard deviation of the experimental data remains within ±3.2 dB over the entire frequency range, confirming good repeatability of the measurement. The overall trend and the location of the attenuation feature are broadly similar, the accelerometer mass-loading effect is the primary factor contributing to the frequency difference observed. This frequency range corresponds to the low-order dispersion branches shown in Figure 11, suggesting that the one-dimensional Bloch model provides a useful qualitative reference for interpreting the low-frequency response trend of finite structures. It should be noted, however, that the Bloch model describes infinite periodic structures, whereas the experimental specimen consists of only four unit cells. Therefore, the observed transmissibility decrease near 65–70 Hz is interpreted as a finite-structure response trend rather than a strict bandgap predicted by the infinite-period Bloch analysis.

### 5.5. Regulation of Low-Order Propagation Characteristics by Structural Parameters

Based on the validation of the low-order wave propagation characteristics of the baseline configuration, the influence of structural parameters on the low-order propagation frequencies is further investigated. In this study, the curved-rod cross-sectional size and nodal attached mass are selected as two representative tuning parameters, corresponding to changes in the equivalent stiffness and nodal inertia, respectively.

#### 5.5.1. Effect of the Curved-Rod Cross-Sectional Size

Table 7 summarizes the equivalent stiffnesses and coupling indices for different cross-sectional sizes. As the rod diameter increases, the axial, torsional, and coupling stiffnesses all increase, confirming that the cross-sectional size governs the overall stiffness level of the unit cell and thus provides a direct geometric parameter for tuning the low-order dispersion frequencies.

**Figure 16:**
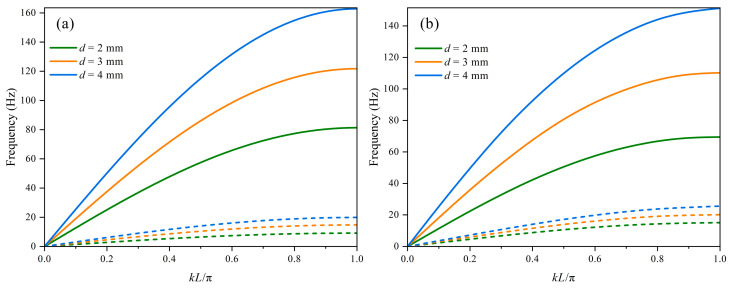
Dispersion curves for different cross-sectional diameters: (**a**) analytical prediction; (**b**) Bloch finite element result. Solid and dotted lines denote Branch 1 and Branch 2, respectively.

Figure 16 compares the theoretical dispersion curves with the Bloch finite element results for different cross-sectional sizes. Both results show that increasing the curved-rod cross-sectional size shifts the two low-order dispersion branches toward higher frequencies. This indicates that the cross-sectional size increases the low-order propagation frequencies of the periodic structure mainly by enhancing the equivalent stiffness of the unit cell. Although certain numerical discrepancies exist between the theoretical and finite element results, they exhibit consistent frequency variation trends.

#### 5.5.2. Effect of Nodal Attached Mass

The effect of nodal inertia on the low-order propagation frequencies is analyzed by varying the equivalent nodal mass and mass moment of inertia about the central axis while keeping the equivalent stiffness matrix unchanged. The attached mass is placed at the nodal end-plate position, so it does not alter the rod centerline geometry or the equivalent stiffness, but modifies the nodal inertial parameters in the periodic chain model. The equivalent mass and mass moment of inertia under different attached-mass conditions are listed in Table 8.

**Figure 17:**
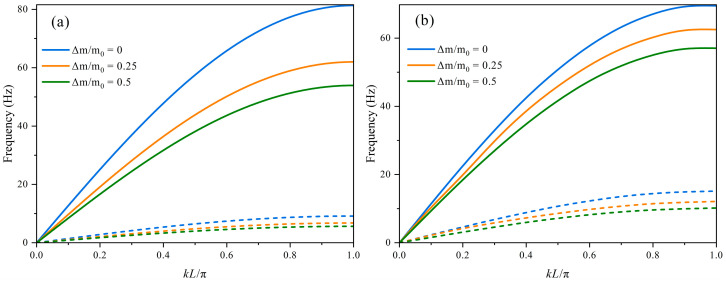
Dispersion curves under different attached mass ratios: (**a**) theoretical prediction; (**b**) finite element result. The solid line and the dotted line respectively represent Branch 1 and Branch 2.

Figure 17 shows the low-order dispersion curves under different attached-mass conditions, obtained by substituting the updated nodal inertial parameters into the one-dimensional Bloch model. As the attached mass increases, both low-order branches shift toward lower frequencies. Unlike stiffness-level adjustments (e.g., changing the rod cross-sectional size), attached mass modifies only the inertial parameters without altering the equivalent stiffness matrix, making it more suitable for secondary frequency tuning after fabrication, whereas cross-sectional adjustment belongs to stiffness-level regulation during the design stage.

The one-dimensional Bloch model captures the main trends of the low-order dispersion branches and finite-chain transmissibility responses. The compression–torsion coupling stiffness alters the frequency positions and modal compositions of the dispersion branches, and the corresponding frequency range exhibits attenuation in the acceleration transmissibility. The low-frequency response can be tuned by modifying the curved-rod cross-sectional size to adjust the axial, torsional, and coupling stiffnesses, or by adding nodal mass to increase translational inertia and shift characteristic frequencies downward. These results demonstrate that low-frequency regulation can be achieved through geometric stiffness tuning and nodal-inertia tuning without introducing additional local resonators or complex auxiliary mechanisms.

### 5.6. Discussion

#### 5.6.1. Comparison with Recent Compression–Torsion Coupled and Wave-Regulating Metamaterials

To clarify the novelty and scope of the present framework, A comparison of representative periodic structures frameworks is presented in Table 9, focusing on differences in degrees of freedom, kinematic assumptions, inertia representations, and wave propagation methods with the present work. In contrast, the present work uses the equivalent stiffness matrix of a spatial curved-rod unit cell as a bridge between static compression-induced rotation and low-order wave propagation in a periodically assembled structure.

The comparison highlights differences in how compression–torsion coupling is modeled in periodic structures. Existing studies mainly utilize such coupling for static functionalities, while dynamic behavior is typically achieved through resonance or inertia-based mechanisms. In contrast, the present work shows that the compression–torsion stiffness matrix of spatial curved rods influences both static coupling and low-order dynamic dispersion. Compared with conventional inertia- or resonance-based approaches, the proposed framework enables stiffness-driven tuning of low-frequency wave behavior in periodic structures.

#### 5.6.2. Advantages and Limitations

The main advantage of the proposed structure lies in its continuous spatial curved-rod architecture. The smooth winding rods provide an integrated axial–rotational load-transfer path without discrete hinges, local resonators, or external inertial devices. The circumferentially distributed layout helps maintain structural balance, while the monolithic configuration is suitable for additive manufacturing and periodic assembly. In addition, the curved-rod centerline provides explicit geometric parameters for tuning the equivalent stiffness and compression–torsion coupling response.

The present study also has several limitations. The theoretical model is based on small-deformation assumptions and retains only the axial displacement and rotation about the central axis as generalized degrees of freedom. The curvature and geometric torsion increments are treated as centerline-based reduced-order measures rather than complete material-frame rod strains. In addition, the experimental transmissibility is obtained from a finite specimen composed of four repeated unit cells and may be affected by boundary conditions, damping, manufacturing deviations, installation conditions, and sensor mass loading. These limitations define the applicable scope of the present reduced-order model but do not change the main finding that the spatial curved-rod geometry introduces compression–torsion coupling and affects the low-order dynamic response of the periodic structure. Future work may incorporate machine-learning-assisted design and material-level optimization, such as nanoparticle-reinforced materials [48], to further enhance stiffness, fracture resistance, damping capacity, and durability.

## 6. Conclusions

This study presents a compression–torsion coupled periodic structure based on a spatial curved-rod unit cell. The static coupling behavior, low-order wave propagation characteristics, and finite-chain dynamic response were investigated through theoretical analysis, finite element simulations, and experimental validation. The main conclusions are as follows.

(1) The proposed spatial curved-rod unit cell exhibits a stable compression-induced rotational response within the small-deformation regime. A two-degree-of-freedom equivalent stiffness matrix is established, providing a unified representation of the axial, torsional, and compression–torsion coupling stiffnesses governing the static response.
(2) The reduced-order equivalent model reasonably predicts the axial and torsional stiffnesses and captures the overall trend of the coupling stiffness. The experimentally validated free-torsion coupling coefficient further demonstrates an approximately linear relationship between axial compression and end-plate rotation, confirming the effectiveness of the proposed equivalent formulation.
(3) Compression–torsion coupling stiffness plays a key role in governing the frequencies and modal compositions of the two lowest dispersion branches. The reduced-order Bloch model successfully reproduces the qualitative low-frequency wave propagation characteristics, while finite-chain transmissibility measurements verify the predicted dynamic response. In addition, the low-frequency response can be effectively tailored by varying the rod cross-sectional size and the attached nodal mass.

## Figures and Tables

**Table 1:** Main parameters used for the analytical, numerical, and experimental models.

Category	Parameters	Symbol	Value
Geometry	Cylindrical radius of rod centerline	R	22.5 mm
	Initial unit-cell height	$H_{0}$	70 mm
	Rod diameter in baseline model	d	2 mm
	End-plate radius and thickness	Rp, Hp	25 mm, 2.5 mm
	period length	L	75 mm
Material	Young’s modulus	E	0.718 GPa
	Poisson’s ratio	ν	0.38
	Shear modulus	G	0.260 GPa
	Density	ρ	$1050\;kg/m^{3}$
Inertia	Equivalent nodal mass	m	$4.866\times 10^{-3}\;kg$
	Equivalent polar rotational inertia	Jm	$2.48\times 10^{-6}\;kg\cdot m^{2}$
Loading	Static loading	−	axial displacement loading
	Dynamic excitation	−	sinusoidal displacement excitation
Numerical	Finite element mesh	−	rods: 0.5 mm; plates: 2.0 mm
	Boundary condition	−	Left sinusoidal excitation, right free

**Table 2:** Mesh convergence study for the baseline configuration (d=2 mm): stiffness parameters and Bloch eigenfrequencies at the Brillouin zone boundary (kL/π=1).

Mesh	Max Element Size (Rods/Plates)	DOFs	$K_{h}$ (N/m)	$K_{c}$ (N)	$K_{m}$ (N·m/rad)	$f_{1}$ (Hz)	$f_{2}$ (Hz)	Solution Time
1 (coarse)	1.0 mm/4.0 mm	36,228	3.90 × $10^{4}$	3.85 × $10^{2}$	5.83	15	73	219 s
2 (baseline)	0.5 mm/2.0 mm	142,998	4.10 × $10^{4}$	4.07 × $10^{2}$	6.09	16	82	226 s
3 (fine)	0.25 mm/1.0 mm	590,246	4.18 × $10^{4}$	4.15 × $10^{2}$	6.22	18	85	846 s

**Table 3:** Analytical and finite element estimates of the effective stiffness parameters of the unit cell.

Parameters	Organization	Theoretical Prediction	FE Extraction Method	FE Result	Relative Error
$K_{h}$	N/m	$4.36\times 10^{4}$	F−u	$4.10\times 10^{4}$	5.9%
$K_{c,u}$	N	$4.83\times 10^{2}$	M−u	$4.07\times 10^{2}$	15.7%
$K_{c,\phi }$	N	$4.83\times 10^{2}$	F−ϕ	$4.06\times 10^{2}$	15.9%
$K_{m}$	N·m/rad	5.63	M−ϕ	6.09	8.1%

**Table 4:** Deviation between the assumed-field centerline and the FE-extracted centerline under representative loading cases and rod diameters.

Loading Case	Load Level	Sampling Points	$E_{RMS}/L$ (%)	$E_{max}/L$ (%)	$E_{r,max}/R$ (%)
Axial displacement, d = 2 mm	u=2 mm, ϕ=0	20	4.6%	6.3%	8.1%
End rotation, d = 2 mm	ϕ=0.1 rad, u=0	20	3.8%	4.2%	7.7%
Axial displacement, d = 3 mm	u=2 mm, ϕ=0	20	4.9%	5.2%	6.1%
End rotation, d = 3 mm	ϕ=0.1 rad, u=0	20	4.1%	4.3%	5.9%

**Table 5:** Analytical, finite element, and experimental estimates of the effective axial stiffness and free-torsion coupling coefficient.

Parameters	Theoretical Prediction	FE Result	Experimental Result	Relative Error (Theory/FE)	Relative Error (FE/Experiment)
$K_{h}(N/m)$	$4.36\times 10^{4}$	$4.10\times 10^{4}$	$3.93\times 10^{4}$	9.86%	4.14%
η(rad/m)	85.7	73.3	70.9	14.46%	3.2%

**Table 6:** Comparison of errors between analytical dispersion predictions and Bloch finite element results.

Branch	Average Error/Hz	Average Relative Error	The Position of the Maximum Error
Branch 1	+6.6	14.7%	kL/π≈1
Branch 2	−3.8	39.9%	kL/π≈1

**Table 7:** Effective stiffness parameters and compression–torsion coupling indices for different cross-sectional sizes.

Configuration	Cross-Sectional Diameter	*K_h_* (N/m)	*K_c_* (N)	*K_m_* (N·m/rad)	*η* (rad/m)	*χ*
1	2 mm	$4.36\times 10^{4}$	$4.83\times 10^{2}$	5.63	85.7	0.9748
2	3 mm	$9.82\times 10^{4}$	$1.08\times 10^{3}$	12.6	85.7	0.9709
3	4 mm	$1.74\times 10^{5}$	$1.93\times 10^{3}$	22.5	85.7	0.9754

**Table 8:** Effective inertial parameters for different added mass ratios.

Configuration	Additional Mass Ratio Δ*m*/*m*_0_	m (kg)	J (kg⋅m^2^)
1	0	$4.866\times 10^{-3}$	$2.48\times 10^{-6}$
2	0.25	$6.082\times 10^{-3}$	$3.24\times 10^{-6}$
3	0.5	$7.299\times 10^{-3}$	$4.01\times 10^{-6}$

**Table 9:** Comparison between representative metamaterial modeling frameworks and the present work.

Framework	DOF	Kinematic	Inertia	Wave Method	Coupling Mechanism	Key Characteristic
Chiral/ anti-chiral [6]	Translational + rotational	Geometric lattice coupling	Distributed/FE mass	Bloch/FE	Geometry- induced coupling	Geometry-driven response
Origami [32]	Hinge rigid DOF	Folding constraints	Lumped hinge mass	Bloch/FE	Reconfiguration coupling	Nonlinear geometry
Inertial Amplification [26]	Translational inertial DOF	Linkage amplification	Added mass/ inerter	Transfer matrix/Bloch	Inertia-driven coupling	Inertia-based bandgaps
Beam lattices [24]	Axial + bending DOF	Beam theory	Distributed mass	Bloch	Periodic stiffness	Bragg scattering
Present work	Axial + rotation	Reduced-order centerline model	Reduced lumped mass	Reduced Bloch model	Stiffness-induced coupling	Stiffness → dispersion mapping

## Data Availability

The original contributions presented in this study are included in the article. Further inquiries can be directed to the corresponding author.
